# Overexpression of WRAP53 Is Associated with Development and Progression of Esophageal Squamous Cell Carcinoma

**DOI:** 10.1371/journal.pone.0091670

**Published:** 2014-03-13

**Authors:** Xuguang Rao, Daofu Huang, Xuxia Sui, Gefei Liu, Xuhong Song, Jinglian Xie, Dongyang Huang

**Affiliations:** 1 Department of Thoracic and Cardiovascular Surgery, Second Affiliated Hospital of Shantou University Medical College, Shantou, Guangdong, China; 2 Department of Thoracic Surgery, Affiliated Cancer Hospital of Guangzhou Medical University, Guangzhou, Guangdong, China; 3 Key Laboratory of High Cancer Incidence Coastal Chaoshan Area of Guangdong Higher Education Institutes, Department of Cell Biology, Shantou University Medical College, Shantou, Guangdong, China; National Cancer Institute, National Institutes of Health, United States of America

## Abstract

**Background:**

Esophageal squamous cell carcinoma (ESCC) is a highly aggressive cancer whose underlying molecular mechanisms are poorly understood. The natural antisense transcript (NAT) *WRAP53* regulates *p53* expression and WRAP53 protein is a component of telomerase. NATs play key roles in carcinogenesis, and although WRAP53 is known to increase cancer cell survival, its role in ESCC clinicopathology is unknown. The aim of this study was to investigate WRAP53 expression in ESCC and to correlate it with clinicopathological characteristics.

**Methods:**

WRAP53 mRNA and protein expression was measured by quantitative PCR (qRT-PCR) and western blotting, respectively, in 4 ESSC cells lines and in 45 paired ESCC and non-neoplastic esophageal mucosa tissues. To correlate WRAP53 protein expression with clinicopathological characteristics, immunohistochemistry (IHC) was performed on 134 ESCC and 85 non-neoplastic esophageal mucosa tissues.

**Results:**

Expression of WRAP53 was detected in all ESCC cell lines and was upregulated in the ESCC tissues compared with the corresponding non-neoplastic tissues (P<0.01). More cells expressed WRAP53 protein in the ESCC tissues than in the non-neoplastic tissues (P<0.01). Overexpression of WRAP53 was significantly correlated with tumor infiltration depth (P = 0.000), clinical stage (P = 0.001), and lymph node metastasis (P = 0.025). Wrap53 expression was not correlated with age, gender, or tumor differentiation.

**Conclusion:**

This report indicates increased expression of WRAP53 in ESCC and that WRAP53 overexpression is correlated with tumor progression. WRAP53 may play a significant role in ESCC; accordingly, WRAP53 could be a useful biomarker for ESCC.

## Introduction

Natural antisense transcripts (NATs), also called antisense RNAs, are RNAs that contain sequences that are complementary to other endogenous transcripts. Antisense RNAs may also encode proteins or may exist only as non-protein-coding transcripts [1], [2]. In recent years, investigations into NAT functions have indicated that NATs play key roles in carcinogenesis and the development of cancers [3]–[9]. The tumor suppressor gene TP53 is the most frequently mutated gene in human cancers [10]. P53 is a pivotal tumor suppressor that induces apoptosis, cell-cycle arrest, and senescence in response to stress signals such as DNA damage, hypoxia, or activated oncogenes [11], [12]. A natural antisense transcript to *p53* (WRAP53) has recently been identified; WRAP53 gives rise to p53 antisense transcripts that regulate p53 mRNA expression and are required for p53 activity upon DNA damage [13]. *WRAP53* transcripts may also be translated into WRAP53 protein, supporting the proliferation of progenitor cells and tumor cells by binding to telomerase to add telomere repeats to chromosome ends [14], [15].

Esophageal cancer (EC) is one of the most common malignant tumors, resulting in poor prognosis worldwide [16]. ESCC is the most frequent histological EC subtype, accounts for more than 90% of ECs, and results in clinical outcomes with high mortality rates in China [17], [18]. Esophageal carcinogenesis involves multiple cellular alterations, including aberrant cell cycle control, DNA repair, cellular enzymes, and growth factor and nuclear receptors [16]. To reduce mortality and improve the success of therapies, many studies have focused on identifying biomarkers for early-stage ESCC detection and on putting these markers to clinical use [19]. P53 protein accumulation is an important early biomarker for identifying high-risk subjects for EC [20]. As a p53 NAT, *WRAP53* regulates endogenous p53 mRNA levels and therefore has a critical role in p53 function. Overexpression of WRAP53 increases p53 mRNA and protein levels [13]. Most NATs are non-coding and exert their function only at the RNA level. However, *WRAP53* mRNA also encodes WRAP53 protein (alternatively described as WDR79 or TCAB1), which has been identified as essential for Cajal body maintenance by binding and directing small Cajal body-specific RNAs (scaRNAs) to the Cajal bodies [14], [21]. Downregulation of WRAP53 expression can induce cell death by apoptosis [13]. However, the role of WRAP53 in tumor development and progression remains largely unclear, and its correlation with clinical significance remains to be elucidated.

In this study, we investigated the expression of WRAP53 protein and mRNA in EC cell lines, ESCC tumors, and adjacent non-neoplastic esophageal mucosa tissue. Our work indicates that WRAP53 is overexpressed in ESCC tissue compared to adjacent non-neoplastic esophageal mucosa tissue and that WRAP53 expression closely correlates with the clinicopathology in ESCC patients.

## Patients and Methods

This study was approved by the Ethics Committee of Shantou University Medical College. During this study, informed consent in writing was obtained from each patient and the study protocol conformed to the ethical guidelines of the 1975 Declaration of Helsinki as reflected in a priori approval by the Ethics Committee of Shantou University Medical College.

### Cell lines and cell culture conditions

The esophageal carcinoma cell lines KYSE150 and KYSE180 were kindly provided by professor Liyan Xu [22] (Department of Biochemistry and Molecular Biology, Shantou University Medical College) who originally obtained these cells lines from JCRB Cell Bank and Dr. Yutaka Shimada’s lab [23]. The EC109 and EC9706 cell lines were kindly provided by professor Xuhong Song [24] (Department of Biochemistry and Molecular Biology, Shantou University Medical College). All cell lines were cultured in RPMI 1640 supplemented with 10% fetal bovine serum (FBS) at 37°C under a 5% CO2 atmosphere.

### Tissues and patient histories

Tissues were obtained from 134 patients (108 males and 26 females; median age, 51.6 ± 8.5years; range, 32–76 years) who had undergone radical esophagectomy in the Department of Thoracic and Cardiovascular Surgery, Second Affiliated Hospital of Shantou University Medical College (Shantou, Guangdong, China) from 30 March, 2008, to 15 July, 2011. Forty-five pairs of samples were preserved in liquid nitrogen immediately after collection for subsequent testing. Each sample was matched with the adjacent non-neoplastic mucosa removed during the same surgery, usually 5–10 cm away from the periphery of the main tumor lesion. All patients were selected at their first diagnosis and none had received radiotherapy, chemotherapy, and/or immunotherapy before the esophagectomy. All ESCC and adjacent non-neoplastic mucosa tissues were independently confirmed by two pathologists who were blinded to the original diagnosis. For this examination, strict criteria were used to diagnose the non-neoplastic mucosa tissue as having no carcinoma, dysplasia, or atypical hyperplasia; however, chronic inflammation was allowed for inclusion. We collected clinical data, including gender and age of patients, depth of tumor invasion, cell differentiation, lymph node metastasis, and clinical tumor-node-metastasis (TNM) stage. Primary tumor staging followed the seventh edition of the TNM staging system of the American Joint Committee on Cancer (AJCC). Depth of infiltration was classified into four groups as follows: pT1 to submucosa, pT2 to muscularis propria, pT3 to adventitia, and pT4 to adjacent structures [25].

### Immunohistochemical staining

Immunohistochemical staining with anti-WRAP53 antibody (1:150 dilution, Proteintech group, Chicago, USA) was performed as follows: tissues were fixed in 10% formaldehyde, embedded in paraffin, cut into 4-μm sections, and mounted on slides. Slides were deparaffinized, rehydrated, and antigen unmasking was processed in 0.01 M sodium citrate buffer (pH 6.0) at high temperature (120°C) for 5 min, cooled at room temperature for 30 min, and immersed in 3% hydrogen peroxide solution for 10 min. Slides were washed twice in PBS, blocked with 10% normal goat serum at 37°C for 30 min, and then incubated with rabbit polyclonal WRAP53 antibody overnight at 4°C. After washing with phosphate-buffered saline (PBS), the sections were treated with corresponding streptavidin peroxidase–conjugated secondary antibody. Diaminobenzidine (DAB) was used to visualize WRAP53 antibody binding, and the tissue sections were counterstained with hematoxylin. Primary antibody was replaced by PBS in the negative control.

### Slide evaluation of immunohistochemical staining

Immunostaining for WRAP53 was graded by a semiquantitative method on a scale that took into account the intensity and distribution of the staining. WRAP53 immunostaining was examined by two pathologists using light microscopy. WRAP53 expression was determined from at least 1,000 cells that were systematically counted at ×400 magnification in five visual fields. In the immunohistochemistry (IHC) assay for WARP53, the presence of nuclear staining was considered to be significant. Nuclear staining was detected in all tissue samples examined, whereas cytoplasmic staining was detected in some, but not all tumor samples. We therefore measured and quantified WRAP 53 staining within the nucleus. Staining intensity was graded as 0 (negative), 1 (weak), 2 (moderate), and 3 (strong); the percentage of positive cells examined was scored as 0 (no positive cells), 1 (< 10%), 2 (11–50%), 3 (51–80%), and 4 (> 80%). The two scores were multiplied and the immunoreactive score (IRS; values ranging from 0–12) was determined: 0 (negative), 1–3 (weak), and 4–6 (positive); multiplication values of 8, 9, and 12 were scored as strongly positive [26]. A score of ≥ 4 points was considered positive WRAP53 expression.

### RNA extraction and quantitative real-time RT-PCR

Total RNA was isolated from 45 pairs of frozen tissue samples (from the 134 pairs of ESCC and their corresponding non-neoplastic esophageal mucosa tissues) using TRIzol reagent (Invitrogen, USA). The concentration and purity of the RNA in each sample was measured by absorbance at 260 and 280 nm using a spectrophotometer. Total RNA was reverse transcribed into single-stranded cDNA by using the RT reagent Kit (TaKaRa, Shiga, Japan). Real-time quantitative PCR was performed with an ABI Prism 7000 (Applied Biosystems) machine and by using Platinum SYBR Green qPCR SuperMix-UDG (Invitrogen, USA). The PCR amplification consisted of the following cycling program: 50°C for 2 min, 95°C for 2 min, followed by 40 cycles of 95°C for 15 s and 60°C for 30 s. The PCR primer sequences were designed according to the human *WRAP53* and *GAPDH* gene sequences reported in the literature and as available in GenBank [13]. The *WRAP53* primers were the following: forward, 5′-TGGCACAAAGCTGGACAGT-3′ and reverse, 5′-GCTGGGTCCTGGTCTGAAG-3′. The GADPH primers were the following: forward, 5′-GCACCGTCAAGGCTGAGAAC-3′ and reverse, 5′-TGGTGAAGACGCCAGTGGA-3′. The specificity of the amplification was confirmed through dissociation curve analysis yielding single peaks from PCR products; 2% agarose gel electrophoresis was used to confirm the correct sizes of the PCR products. *GAPDH* expression was used as an internal control to normalize *WRAP53* expression in the samples. PCR reactions of each sample were conducted in triplicate. The relative expression of *WRAP53* was calculated by the 2^−ΔΔCt^ method (ΔΔCt  = (Ct_WRAP53_-Ct_GAPDH_)_ESCC tissue_-(Ct_WRAP53_-Ct_GAPDH_)_matched non-neoplastic esophageal mucosa tissue._ For the matched non-neoplastic esophageal mucosa tissue control sample, the ΔΔCt was 0 and 2^−ΔΔCt^ was 1.

### Western blot analysis

Tissues selected by the qRT-PCR method were subjected to protein analysis by using western blotting. Frozen tissues were lysed in RIPA buffer (25 mM Tris, pH 7.6, 150 mM NaCl, 1% Nonidet P-40, 1% sodium deoxycholate, and 0.1% sodium dodecyl sulfate) containing a protease-inhibitor cocktail on ice for 30 min. The different groups of esophageal carcinoma cells were washed 3 times with PBS before lysis. The resulting cell lysates were clarified by centrifugation at 12,000 ×*g* for 15 min at 4°C. Proteins from the different groups were separated by 10% SDS–PAGE and transferred to polyvinylidene difluoride membranes (Millipore, Billerica, MA) at 100 V for 120 min on ice. The membranes were blocked with 5% non-fat milk in Tris-buffered saline with Tween 20 (TBST; 100 mM Tris-HCl, pH 7.5, 150 mM NaCl, and 0.1% Tween 20), followed by incubation with a rabbit polyclonal antibody for WRAP53 (1∶1000 in TBST) or rabbit polyclonal antibody for β-actin (1∶2500 in 5% nonfat milk in TBST) overnight at 4°C. After 3 washes in TBST, membranes were exposed to horseradish peroxidase–conjugated secondary antibody (1:3000; Sigma, USA) for 1 h at room temperature. Proteins were detected by enhanced chemiluminescence (Pierce, Rockford, IL USA) and exposed to X-ray film. β-actin was used as a loading control. Protein concentrations were determined with a Gel-pro Analyzer 4.0 (Media CyberneticsInc, USA). WRAP53 protein expression was calculated from the relative intensity ratio of WRAP53 to β-actin protein.

### Statistical analysis

All statistical analyses were performed with SPSS 13.0 for Windows (SPSS Inc, Chicago, USA), and the data were expressed as the mean ± standard deviation (SD). The Wilcoxon test was used to evaluate the statistical significance of the difference in the expression of WRAP53 mRNA and protein. The Chi-square test was used to determine correlations between WRAP53 expression and clinicopathological parameters. In all analyses, a P<0.05 was considered statistically significant.

## Results

### Expression of WRAP53 in ESCC cell lines

**Figure 1:**
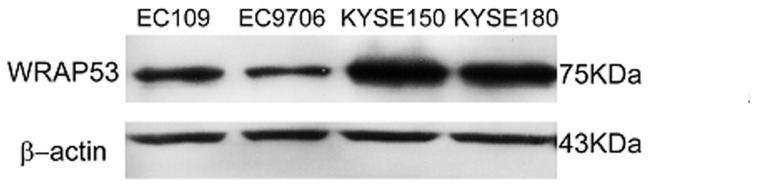
Protein expression of WRAP53 in ESCC cell lines. Western blot of WRAP53 protein expression in four esophageal cancer cell lines; β-actin expression was used as a control.

To investigate the relationship between WRAP53 expression and ESCC, we first examined the expression of WRAP53 in 4 ESCC cell lines (EC109, EC9706, KYSE150, and KYSE180) we detected the expression of WRAP53 protein in all 4 ESCC cell lines with an expected molecular weight of 75 kDa(**Figure 1**).

### WRAP53 is upregulated in ESCC tissues

**Figure 2:**
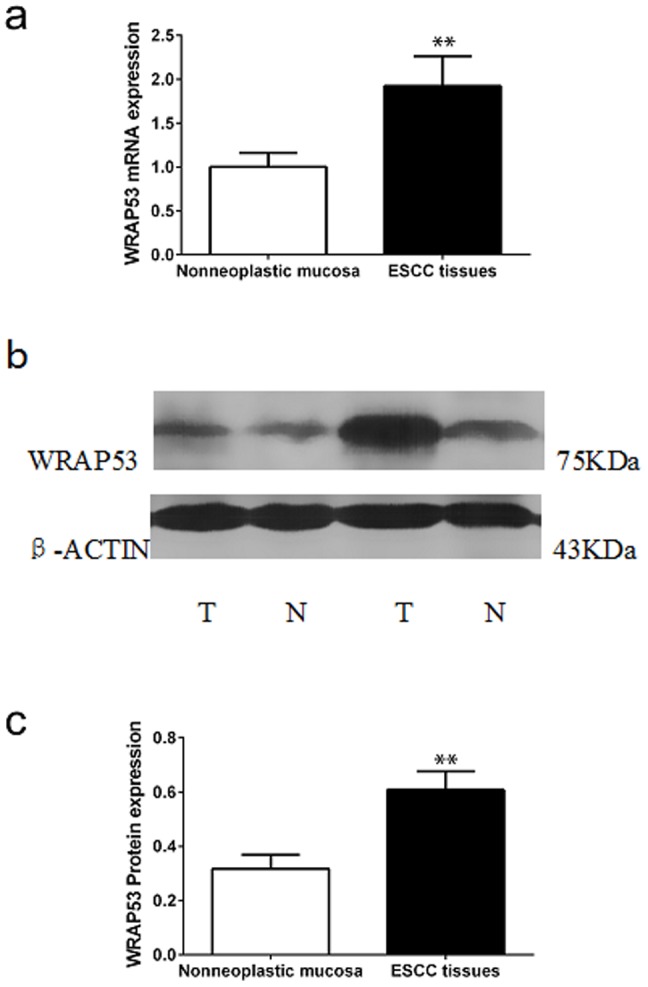
mRNA and protein expression of WRAP53 in ESCC and non-neoplastic tissues. (**a**) Relative expression of WRAP53 mRNA in ESCC tissues and non-neoplastic mucosa. *GADPH* was used as an internal control gene in the qRT-PCR. (**b**) Western blot analysis of WRAP53 protein expression in esophageal carcinoma tissues and non-neoplastic mucosa tissues. Representative blots are shown for the 75-kDa WRAP53 protein. The upper panel is representative of two paired ESCC tissues (marked “T”) and their corresponding non-neoplastic esophageal mucosa tissues (marked “N”); β-actin was used as a control. (**c**) Densitometric values were determined by normalization to β-actin protein levels. **p<0.01.

We further compared *WRAP53* mRNA expression in the ESCC tissues and in the adjacent non-neoplastic esophageal mucosal tissues. *WRAP53* mRNA expression was upregulated in 37/45 (82.2%) of the esophageal carcinoma specimens. The qRT-PCR analysis also showed that the levels of *WRAP53* mRNA expression in ESCC tissues were significantly higher than those in the corresponding non-neoplastic esophageal mucosal tissues (P<0.01) (**Figure 2a**). We next examined WRAP53 protein levels in 45 pairs of ESCC tissues and their adjacent non-neoplastic esophageal mucosal tissues. Results from a representative WB analysis are shown in **Figure 2b**, and a summary of relative WRAP53 protein expression is provided in **Figure 2c**. WRAP53 protein expression was upregulated in 95.6% (43 out of 45) ESCC specimens compared with the corresponding non-neoplastic esophageal mucosal tissue specimens. WRAP53 protein levels were also markedly upregulated in ESCC tissues compared with the non-neoplastic esophageal mucosa tissues (0.61±0.46 vs. 0.32±0.35, P<0.01) (**Figure 2c**).

### Immunohistochemical detection of WRAP53 protein expression in ESCC and adjacent non-neoplastic esophageal mucosa

**Figure 3:**
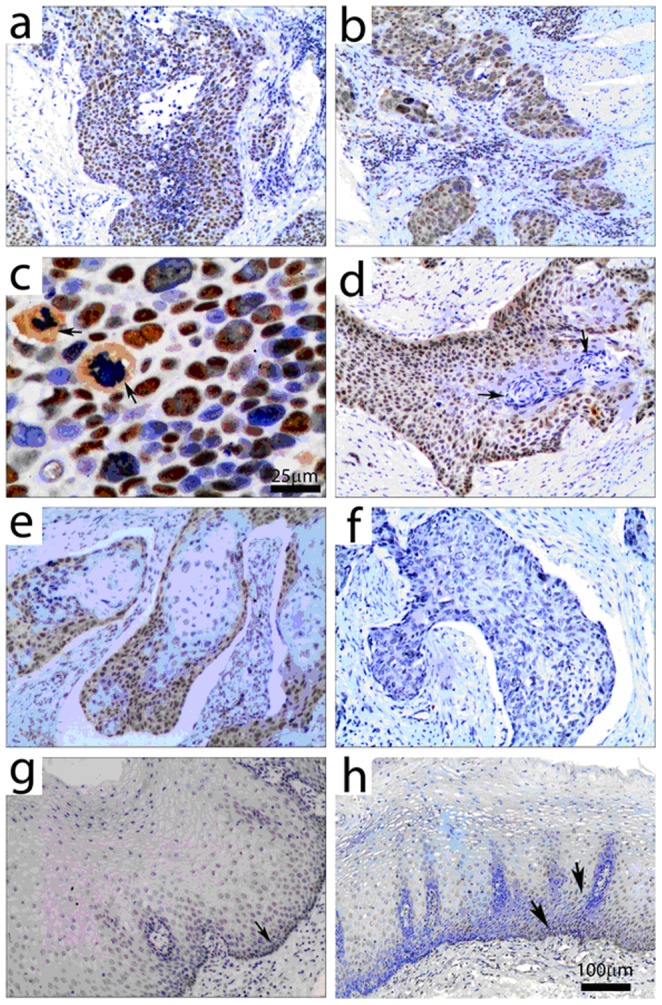
Immunohistochemical detection of WRAP53 protein expression in esophageal carcinoma and in adjacent non-neoplastic esophageal mucosa. (a) WRAP53 protein is visualized by yellow or brownish yellow staining in ESCC tissues. (b) Nuclear WRAP53 expression in ESCC tissues and weak or absent WRAP53 expression in the adjacent muscularis. (c) Strong expression of WRAP53 protein in poorly differentiated ESCC tissue. (d) and (e) Positive expression of WRAP53 in the well-differentiated ESCC tissue. (f) Negative WRAP53 expression in ESCC tissue. (g) and (h) Expression of WRAP53 is weak in adjacent non-neoplastic esophageal mucosa and is limited in basal and/or suprabasal layer cells. Scale bar  = 25 micron in c and 100 micron in all other figures.

Expression of WRAP53 protein in ESCC tissues (**Figure 3a–f**) and adjacent non-neoplastic esophageal mucosa tissues (**Figure 3g and h**) was analyzed in more detail by IHC. Representative ESCC tissues with positive staining (brown in **Figure 3a–e**) and negative staining (**Figure 3f**) are shown. WRAP53 expression in ESCC tissues was more distinct than in the surrounding tissues including non-neoplastic esophageal mucosa, stroma, and muscularis (**Figure 3b**). Poorly differentiated ESCC tissues showed strongly upregulated WRAP53 expression and WRAP53 was predominantly expressed in the nuclei (arrow in **Figure 3c**). WRAP 53 staining was also observed in the nuclei of cells undergoing pathological mitosis and fully keratinized tumor cells in keratin pearls showed inconspicuous or absent WRAP53 immunoreactivity (arrows in **Figure 3d**). Overall, in well-differentiated nests of carcinomas, the peripheral cells of neoplastic nests were intensely stained, with decreasing immunoreactivity toward the center of the neoplastic nests (**Figure 3e**). In non-neoplastic esophageal mucosa tissues, WRAP53 was also predominantly expressed in the nucleus of epithelial cells. Staining of non-neoplastic esophageal mucosa tissues was mostly weak or absent, with nuclear staining present in basal and suprabasal layer cells (arrows in **Figure 3g and h**).

### Relationship between WRAP53 expression and clinicopathological characteristics of esophageal carcinomas

The association between WRAP53 protein expression and clinicopathological features of esophageal carcinomas was also analyzed (**Table 1**). In 72.4% (97 out of 134) of the ESCC tissues, WRAP53 was higher than in non-neoplastic esophageal mucosa tissues. Overexpression of WRAP53 significantly correlated with the overall frequency of ESCC (P<0.001, **Table 1**). The expression of WRAP53 protein was negatively correlated with the degree of tumor differentiation (P = 0.133). On the other hand, WRAP53 expression was positively correlated with the depth of tumor invasion (P = 0.000) and lymph node metastasis (P = 0.025). The clinical stage of ESCC in patients was classified into stage I to IV according to the TNM classification. Stage I and II tumors showed significantly lower percentages of WRAP53-positive cells compared to stage III and IV tumors (P = 0.001). No statistically significant relationship was observed between WRAP53 expression and gender or age (P > 0.05).

**Table 1:** Relationship between WRAP53 expression and clinicopathological characteristics of esophageal carcinomas in patients.

Variable	Patients (N)	Wrap53 expression		?^2^	P value
		+	–		
Overall frequency					
ESCC	134	97(72.4%)	37 (27.6%)	45.335	0.000
Nonneoplastic	85	22 (25.9%)	63 (74.1%)		
Age (years)					
<60	81	59 (72.8%)	22 (27.2%)	0.021	0.886
≧60	53	38 (71.7%)	15 (28.3%)		
Gender					
Male	108	78 (72.2%)	30(27.8%)	0.008	0.931
Female	26	19 (73.1%)	7(26.9%)		
Pathological differentiation grade					
Well	56	36 (64.3%)	20 (35.7%)	3.173	0.133
Moderately	56	44 (78.6%)	12 (21.4%)		
Poorly	22	17 (77.3%)	5 (22.7%)		
T stage					
T1+T2	39	18 (46.2%)	21 (53.8%)	18.942	0.000
T3+T4	95	79 (83.2%)	16 (16.8%)		
Lymph node metastasis					
Negative	77	50(64.9%)	27 (35.1%)	5.031	0.025
Positive	57	47 (82.5%)	10 (17.5%)		
Clinical stage					
I+II	83	52 (62.7%)	31 (37.3%)	10.345	0.001
III+IV	51	45 (88.2%)	6 (11.8%)		

## Discussion

Esophageal cancer (EC) is one of the most common malignant diseases worldwide. The processes during EC carcinogenesis and progression involve complex factors, stages, and changes at the molecular level [16], [18], [19]. Recent studies of NATs have indicated that NATs have a close correlation with carcinogenesis and the development of cancer. WRAP53 has previously been shown to be an antisense transcript that regulates the p53 tumor suppressor. Expression of *WRAP 53α* and *p53* transcripts have been detected in a variety of human tumor cell lines (for example, in U2OS, HCT116, U87, MCF-7, and HEK293 cells) [13]. Mahmoudi and colleagues recently demonstrated that WRAP53 protein is overexpressed in many different cancer cell lines, and that WRAP53 overexpression promotes cellular transformation [27]. In this study, we determined WRAP53 expression at protein levels in cancer cell lines of identical origin [23], [28], [29]. WRAP53 expression in 4 ESCC cell lines was examined using western blot, and expression of WRAP53 protein was detected in all of these ESCC lines.

The WRAP53 gene has been shown to be involved in the development of primary human cancer. Schildkraut et al. have found that some single-nucleotide polymorphisms (SNPs) in WRAP53 modestly increase the risk of serous and endometrioid invasive ovarian cancer [30]. SNPs in WRAP53 are found to be overrepresented in women with breast cancer, especially in estrogen receptor–negative breast cancer [31]. Here, we observed significantly higher expression of WRAP53 mRNA and protein in ESCC tissues than in the paired non-neoplastic mucosa tissues. About 96% of the ESCC tissues had greater levels of WRAP53 protein expression compared with the non-neoplastic esophageal mucosa tissues, and *WRAP53* mRNA expression was upregulated in 82% of the ESCC patients. Taken together, these results suggest that WRAP53 may act as an oncogene in ESCC. WRAP53 protein expression in EC tissues was significantly higher than that in non-neoplastic esophageal mucosa tissues (P<0.001). As far as we know, this is the first report showing a difference of WRAP53 expression in cancerous and non-cancerous tissues. Therefore,WRAP53 expression could be involved in ESCC development.

We also characterized the expression pattern of the WRAP53 protein by using (IHC). The IHC results showed that, in general, WRAP53 was predominantly expressed in the nuclei of tumor cells, especially in immature tumor cells and progenitor cells in the basal and/or suprabasal layer. These immunohistochemical studies suggest that WRAP53 might be involved in the proliferation of ESCC. Investigations with samples from patients with head and neck squamous cell carcinoma showed that WRAP53 levels are higher in patients with recurrent tumors compared with patients with positive cancer outcomes [27]. Our study of the tissues of 134 ESCC patients showed that increased WRAP53 protein expression correlates with increased depth of tumor invasion and that WRAP53 expression is significantly higher in T III and T IV ESCC tissues than in TI and T II ESCC tissues. We also observed that overexpression of WRAP53 is significantly correlated with lymph node metastasis and TNM stage. Therefore, the overexpression of WRAP53 protein appears to play an important role in the progression of ESCC.

In conclusion, our results have shown that the expression of WRAP53, the natural antisense transcript to p53, is significantly upregulated at the level of both mRNA and protein in ESCC tissues compared with non-neoplastic esophageal mucosa tissues. Overexpression of WRAP53 correlated with tumor infiltration depth, clinical stage, and lymph node metastasis. Therefore, WRAP53 may play a significant role in the development and progression of ESCC. Thus, WRAP53 could be a useful biomarker for ESCC and could represent a potential target for treatments of this disease.
